# Current status of *Staphylococcus aureus* infection in a central teaching hospital in Shanghai, China

**DOI:** 10.1186/1471-2180-13-153

**Published:** 2013-07-08

**Authors:** Tianming Li, Yan Song, Yuanjun Zhu, Xin Du, Min Li

**Affiliations:** 1 Department of Laboratory Medicine, Huashan Hospital, Shanghai Medical College, Fudan University, 12 Central Urumqi Road, Shanghai, China

**Keywords:** *Staphylococcus aureus*, Methicillin resistant, Methicillin susceptible, Sequence types, Hospital-acquired infections

## Abstract

**Background:**

To control the spread of methicillin-resistant *Staphylococcus aureus* (MRSA) in hospitals, infection control measures such as hand hygiene practices were introduced into the teaching hospitals in Shanghai, China, in 2008. Currently, there is limited information characterizing the latest hospital-acquired *S. aureus* infections in this area. Therefore, we sought to determine the prevalence, molecular characteristics, and genotype-phenotype correlation of hospital-acquired *S. aureus* infections in Huashan Hospital, one of the largest teaching hospitals in Shanghai.

**Results:**

Among 608 hospital-acquired *S. aureus* clinical isolates obtained from January to December of 2011 in Huashan Hospital, 68.1% were MRSA. The predominant MRSA clones were ST239-SCC*mec*III and ST5-SCC*mec*II. ST239 was mainly recovered from respiratory specimens and sterile body fluids, ST5 was associated with respiratory specimens and blood, and ST1 was most prevalent in urine samples. In this study, 31 dispersed sequence types (STs) of methicillin-susceptible *S. aureus* (MSSA) were identified, most of which caused skin/soft tissue infection and bacteremia. The frequencies of *pvl-*, *muPA-*, and *qacA/B*-positive isolates were 1.6, 9.9, and 11.8% respectively. *MuPA* was more frequently identified in ST1 and ST5, and *qacA/B* was more prevalent in ST239 and ST5. Most of the *pvl***-**positive isolates were MSSA, whereas the majority of *muPA-* and *qacA/B*-positive isolates were MRSA. ST239 and ST5 had higher resistance rates to multiple antibiotics. In Huashan Hospital, the infection rate in the intensive care unit (ICU) was 3.9 per 1000 hospitalized days, but only 1.2 per 1000 hospitalized days in the other wards. Each ward harbored its own dominant STs. Pulsed-field gel electrophoresis showed diversity within the same epidemic *S. aureus* clones originating from the same wards.

**Conclusion:**

There is still a high prevalence of MRSA infections in the teaching hospital in Shanghai. There were also differences in the major infection types caused by MRSA and MSSA, and hospital-acquired *S. aureus* infections in the ICU of Huashan Hospital pose a greater threat to patient safety than in other wards. The high proportion of multiple antibiotic and chlorhexidine-based antiseptic-resistant clones in this hospital underscores the need for more effective infection control measures to help curtail dissemination of MRSA to hospitalized patients.

## Background

*S. aureus* is a globally important human pathogen, causing a variety of diseases such as pneumonia, skin and soft tissue infections, blood-stream infections, osteomyelitis, and endocarditis, as well as toxin-mediated syndromes like toxic shock syndrome and food poisoning [1,2]. Since the onset of the pandemic waves of MRSA over the past decades, it has become the most common cause of both hospital- and community-acquired infection worldwide [3]. According to epidemiological data in 2005, the mean prevalence of MRSA across China was more than 50%, and in Shanghai, the rate was over 80% [4]. To control the spread of MRSA in hospitals, measures such as universal hand hygiene practices have been introduced into Shanghai teaching hospitals. However, as yet there are no programs to screen for asymptomatic MRSA carriers in Chinese hospitals. A re-evaluation of the level of MRSA infection in Shanghai teaching hospitals is required to evaluate the effect of the current infection control measures.

The major MRSA clones that cause infections worldwide belong to five pandemic MRSA lineages: CC5, CC8, CC22, CC30, and CC45 [5-9]. Some virulence genes show strong associations with specific molecular types; for instance, the *sea, sek*, and *seq* genes were identified in all ST239 strains. In contrast, ST5 was strongly associated with the *tst, seg, sei, sem, sen*, and *seo* gene profiles [10]. Conversely, the expression of core genome-encoded virulence genes varies between molecular types of *S. aureus*[11], indicating that discrete sequence types (STs) may correlate with specific infection types. For example, the MRSA clone USA300 mainly causes skin and soft tissue infections (SSTIs) in the United States [12]. To the best of our knowledge, there is limited information characterizing the latest hospital-acquired *S. aureus* infections in hospitals in Shanghai, China. Therefore, we sought to determine the prevalence, molecular characteristics, and genotype-phenotype correlation of hospital-acquired *S. aureus* infections at one of the largest teaching hospitals in Shanghai.

## Results

### The population composition and types of infection caused by *S. aureus*

The clinical and demographic characteristics of the inpatients with *S. aureus* infection are listed in Table 1. Among the 608 hospital-acquired *S. aureus* isolates obtained between January and December 2011, there were 414 (68.1%) MRSA isolates and 194 (31.9%) MSSA isolates. From the clinical medical records, respiratory infection was the most frequently determined infection type caused by *S. aureus*; 67.4% (410/608) of the isolates were from the respiratory tract, and most of the *S. aureus* isolates recovered from respiratory infection were MRSA (78.3%). Conversely, only 36.1% (31/86) of isolates recovered from skin/soft tissue infections were methicillin resistant.

**Table 1:** **Population composition and types of infection caused by *S. aureus***

**Sex**	**No. (%)**	**Age**	**No. (%)**	**Source**	**No. (%)**	**MRSA/MSSA**
Male	401 (66.0%)	14–24	43 (7.1%)	Respiratory	410 (67.4%)	321/89 (78.3%/21.7%)
Female	207 (34.0%)	25–34	56 (9.2%)	Skin/soft tissue	86 (14.1%)	31/55 (36.0%/64.0%)
		35–44	63 (10.4%)	Other sterile body fluids	64 (10.5%)	37/27 (57.8%/42.2%)
		45–54	81(13.3%)	Blood	27 (4.4%)	12/15 (44.4%/55.6%)
		55–64	92 (15.1%)	Urine	21 (3.6%)	13/8 (61.9%/38.1%)
		65–74	96 (15.8%)			
		75–84	72 (11.8%)			
		≥85	105 (17.3%)			

### The STs of current MRSA and MSSA epidemic strains in Huashan Hospital

**Figure 1:**
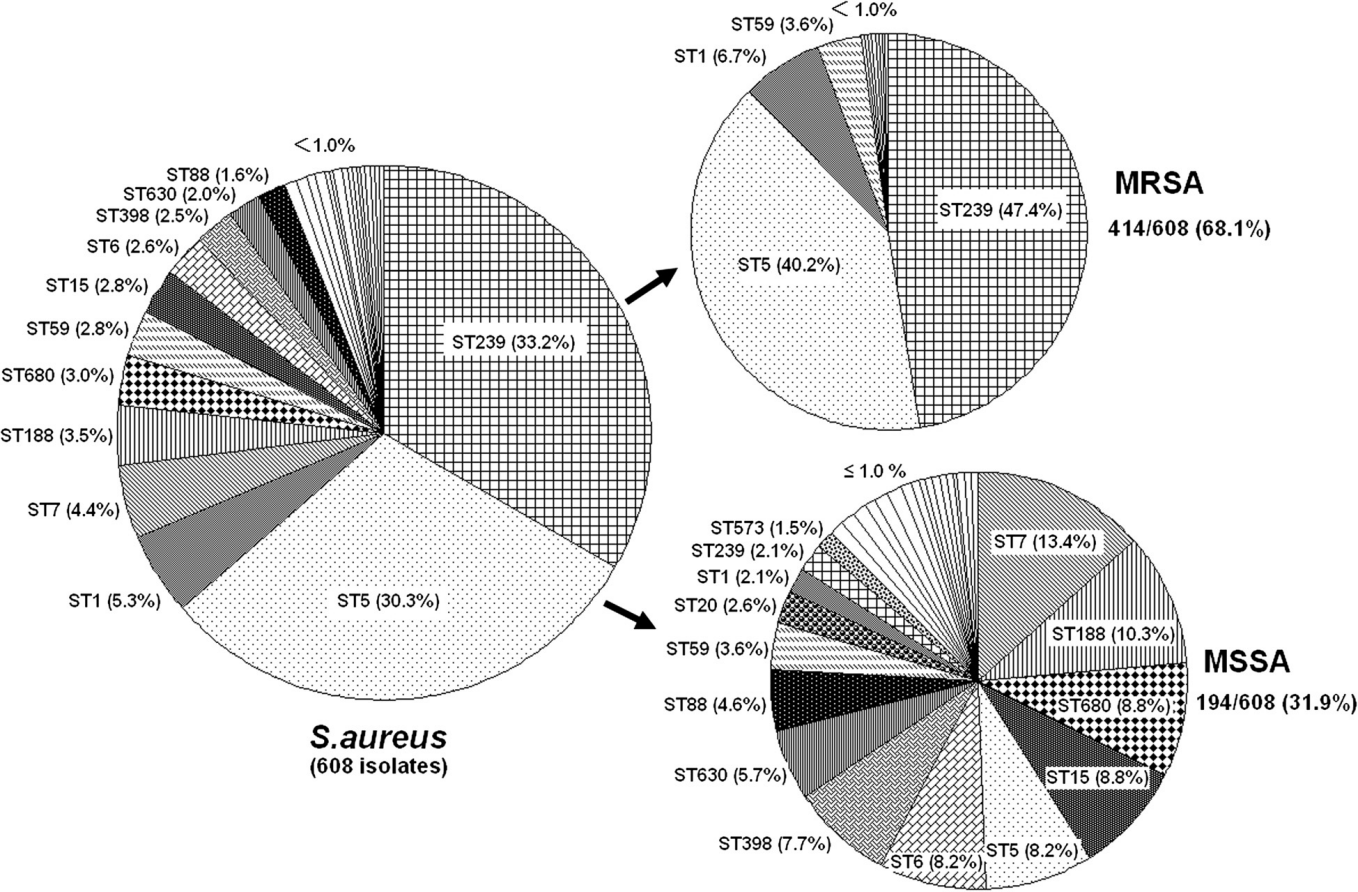
**Molecular types of the 608 non-duplicated *S. aureus *isolates from Huashan Hospital in 2011.**

**Figure 2:**
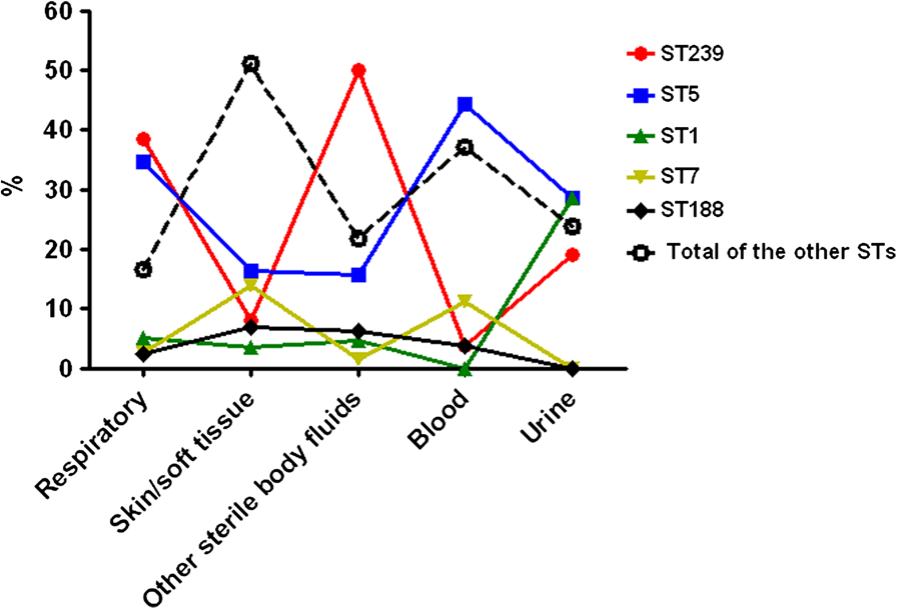
**Prevalence of the epidemic *S. aureus *STs among different clinical specimens.**

All *S. aureus* isolates were typed by multilocus sequence typing (MLST). There were 31 distinct STs identified within the 608 isolates (Figure 1), among which the most frequently represented were ST239 (33.2%, 202/608), ST5 (30.3%, 184/608), ST1 (5.3%, 32/608), ST7 (4.4%, 27/608), and ST188 (3.5%, 21/608). MSSA isolates were associated with all 31 STs, with ST7 (13.4%, 26/194) and ST188 (10.3%, 20/194) being the two dominant types for MSSA isolates. MRSA ST239 was mainly recovered from respiratory specimens (38.5%) and sterile body fluids (50.0%), but was only found at a frequency of 8.1% and 3.7% in skin/soft tissue and blood infections, respectively. ST5 isolates were most often present in respiratory specimens (34.6%) and blood (44.4%), but were isolated at a frequency of only 15.6% and 16.3% from sterile body fluids and skin/soft tissue samples, respectively. ST1 strains were mainly found in urine samples (28.6%). Most other dispersed STs were associated with MSSA strains causing skin/soft tissue infection (51.2%) and bacteremia (37.0%) (Figure 2).

### SCC*mec* types of 414 MRSA isolates from Huashan Hospital

SCC*mec* types I–V were detected in this study. Of the 414 MRSA strains, 0.2% (1/414), 38.9% (161/414), 46.6% (193/414), 12.6% (52/414), and 1.0% (4/414) were SCC*mec* types I–V, respectively. Three MRSA strains carrying SCC*mec* were defined as non-typeable (NT) (Table 2). The predominant STs amongst the MRSA isolates were ST239-SCC*mec*III (43.7%, 181/414) and ST5-SCC*mec*II (35.0%, 145/414). The other two most common MRSA STs were ST1-SCC*mec*IV (6.5%, 27/414) and ST59-SCC*mec*IV(2.2%, 9/414). ST239-SCC*mec*I, ST239-SCC*mec*II, ST5-SCC*mec*III, and ST5-SCC*mec*IV strains were also detected in Huashan Hospital.

**Table 2:** **SCC*mec *types of 414 MRSA isolates arranged by STs**

**MLST**	**MRSA**	**SCC*mec *type**					
	**No.**	**I**	**II**	**III**	**IV**	**V**	**NT**
ST239	198	1 (0.5%)	16 (8.1%)	181 (91.4%)	0	0	0
ST5	168	0	145 (86.3%)	10 (6.0%)	13 (7.7%)	0	0
ST1	28	0	0	1 (3.6%)	27 (96.4%)	0	0
ST59	10	0	0	1 (10.0%)	9 (90.0%)	0	0
ST1821	2*	0	0	0	0	2	0
ST181	1	0	0	0	1	0	0
ST630	1	0	0	0	0	1	0
ST680	1	0	0	0	0	0	1
ST7	1	0	0	0	0	1	0
ST88	1	0	0	0	1	0	0
ST9	1	0	0	0	0	0	1
ST965	1	0	0	0	1	0	0
ST188	1	0	0	0	0	0	1

*STs with less than 10 isolates were not calculated in the percentage of SCC*mec* type.

### Antimicrobial susceptibility profiles

We analyzed 608 *S. aureus* isolates with 31 different STs for antimicrobial resistance (Table 3). All the isolates were susceptible to vancomycin, teicoplanin, and linezolid. Resistance to penicillin (97.4%) was observed most frequently, and ST239 and ST5 strains had significantly higher multiple antibiotic-resistance profiles when compared with other STs. ST5 strains were more susceptible to rifampicin (*P* < 0.001) and sulfamethoxazole + trimethoprim (*P* < 0.001) but more resistant to fosfomycin (*P* < 0.001) than ST239. ST1 isolates were susceptible to most antibiotics except penicillin (96.9%), levofloxacin (59.4%), cefoxitin (87.5%), and cefazolin (78.1%), while ST7 strains were susceptible to most of the antibiotics except penicillin (100.0%), levofloxacin (96.3%), and erythromycin (55.6%). ST188 strains were only resistant to penicillin (90.5%). In this study, 15 isolates of animal infection-associated ST398 were identified, all of which were susceptible to cefoxitin. These isolates were only resistant to penicillin (80.0%) and erythromycin (66.7%).

**Table 3:** **Antimicrobial susceptibility profiles of 608** ***S. aureus *isolates arranged by STs**

**MLST**	**No.**	**P**	**LEV**	**CN**	**FOX**	**CZ**	**E**	**DA**	**RD**	**SXT**	**FOS**	**TEC**	**VA**	**LZD**
		**% Resistance**												
ST239	202	100.0	98.5	98.0	98.0	98.0	85.6	67.3	72.8	23.8	25.3	0.0	0.0	0.0
ST5	184	98.9	91.9	82.1	91.3	91.3	94.0	73.4	3.3	1.1	75.0	0.0	0.0	0.0
ST1	32	96.9	59.4	3.1	87.5	78.1	9.4	3.1	3.1	0.0	18.8	0.0	0.0	0.0
ST7	27	100.0	96.3	18.5	3.7	0.0	55.6	25.9	0.0	0.0	0.0	0.0	0.0	0.0
ST188	21	90.5	4.8	4.8	4.8	4.8	33.3	9.5	0.0	4.8	0.0	0.0	0.0	0.0
ST680	18	100.0	88.9	5.6	5.6	0.0	83.3	0.0	0.0	0.0	0.0	0.0	0.0	0.0
ST59	17	82.4	11.8	0.0	52.9	41.2	82.4	76.5	11.8	5.9	0.0	0.0	0.0	0.0
ST15	17	100.0	0.0	0.0	0.0	0.0	70.6	0.0	0.0	0.0	0.0	0.0	0.0	0.0
ST6	16	100.0	0.0	0.0	0.0	0.0	12.5	0.0	0.0	0.0	0.0	0.0	0.0	0.0
ST398	15	80.0	13.3	20.0	0.0	0.0	66.7	40.0	0.0	0.0	0.0	0.0	0.0	0.0
ST630	12	91.7	50.0	0.0	8.3	0.0	58.3	0.0	0.0	0.0	0.0	0.0	0.0	0.0
ST88	10	90.0	0.0	30.0	10.0	10.0	60.0	30.0	0.0	10.0	0.0	0.0	0.0	0.0
ST20	5^**a**^	5	1	0	0	0	0	0	0	0	0	0	0	0
ST1821	4	4	0	0	2	0	1	0	0	0	0	0	0	0
ST965	3	3	0	1	1	0	3	1	0	0	0	0	0	0
ST573	3	3	1	0	0	0	3	1	0	0	1	0	0	0
ST181	2	2	0	1	1	0	0	0	0	0	0	0	0	0
ST22	2	2	0	0	0	0	2	0	0	0	0	0	0	0
ST25	2	2	0	0	0	0	1	0	0	0	0	0	0	0
ST30	2	2	0	0	0	0	0	0	0	0	0	0	0	0
ST946	2	2	0	0	0	0	1	1	0	0	0	0	0	0
ST338	1	1	0	0	0	0	1	1	0	0	0	0	0	0
ST359	1	1	0	0	0	0	1	0	0	0	0	0	0	0
ST707	1	0	0	0	0	0	1	1	0	0	0	0	0	0
ST223	1	1	1	0	0	0	0	0	0	0	0	0	0	0
ST121	1	1	0	1	0	0	1	1	0	0	0	0	0	0
ST1649	1	1	0	0	0	0	1	0	0	0	0	0	0	0
ST2149	1	0	0	0	0	0	0	0	0	0	0	0	0	0
ST221	1	1	0	0	0	0	0	0	0	0	0	0	0	0
ST9	1	1	0	1	1	1	1	1	0	0	0	0	0	0
ST97	1	0	0	0	0	0	0	0	0	0	0	0	0	0
**Total**	**608**	**97.4**	**72.9**	**60.4**	**68.1**	**66.0**	**75.2**	**51.0**	**25.7**	**8.7**	**32.2**	**0.0**	**0.0**	**0.0**

^a^ STs with less than 10 isolates were not calculated in the percentage of antibiotic resistance.

### Association of genotypes, and detection of the *pvl*, *muPA*, and *qacA/B* genes

Ten (1.6%) isolates were *pvl***-**positive, and they displayed six different STs (Table 4). Four (26.7%, 4/15) *pvl-*positive isolates were ST398, two (20.0%, 2/10) isolates belonged to ST88, and one isolate each belonged to ST338, ST22, ST59, and ST7. With the exception of one ST59 and one ST7 MRSA isolate, all of the *pvl-*positive isolates were MSSA. Sixty (9.9%) isolates were *muPA*-positive, and they displayed 11 different STs. Twenty-one (65.6%, 21/32) *muPA*-positive isolates were ST1, twenty (10.9%, 20/184) isolates belonged to ST5, and seven (3.5%, 7/202) isolates belonged to ST239. There were two isolates each of ST7, ST59, ST630, and ST398, and one isolate belonged to each of the STs ST680, ST188, and ST1036. Most of the *muPA*-positive isolates were MRSA (85.0%, 51/60). Seventy-two (11.8%) isolates were *qacA/B*-positive and displayed eight different STs. Thirty-eight (18.8%, 38/202) and twenty-eight (15.2%, 28/184) *qacA/B*-positive isolates were ST239 and ST5, respectively, while the overall frequency of MRSA-type *qacA/B*-positive isolates was 90.3% (65/72).

**Table 4:** **Association of genotypes, *pvl*, *muPA*, and *qacA/B *genes**

**STs**	**% (No./total)**	**MRSA/MSSA**
***qacA/B*(+)**		
ST239	18.8% (38/202)	100.0% (38/38)
ST5	15.2% (28/184)	96.4% (27/28)
ST6	6.3% (1/16)	0/1
ST7	3.7% (1/27)	0/1
ST20^a^	1/5	0/1
ST22	1/2	0/1
ST1821	1/4	0/1
ST359	1/1	0/1
***pvl*(+)**		
ST398	26.7% (4/15)	0/4
ST88	20.0% (2/10)	0/2
ST59	5.9% (1/17)	1/0
ST7	3.7% (1/27)	1/0
ST338	1/1	0/1
ST22	1/2	0/1
***muPA*(+)**		
ST1	65.6% (21/32)	100% (21/21)
ST630	16.7% (2/12)	0/2
ST398	13.3% (2/15)	0/2
ST59	11.8% (2/17)	2/0
ST5	10.9% (20/184)	100.0% (20/20)
ST7	7.4% (2/27)	0/2
ST680	5.6% (1/18)	1/0
ST188	4.8% (1/21)	0/1
ST239	3.5% (7/202)	7/0
ST1036	1/2	0/1
ST121	1/1	0/1

^a^ STs with less than 10 isolates were not calculated in the percentage of genes present or MRSA/MSSA.

### The prevalence of different genotypes in different wards

**Figure 3:**
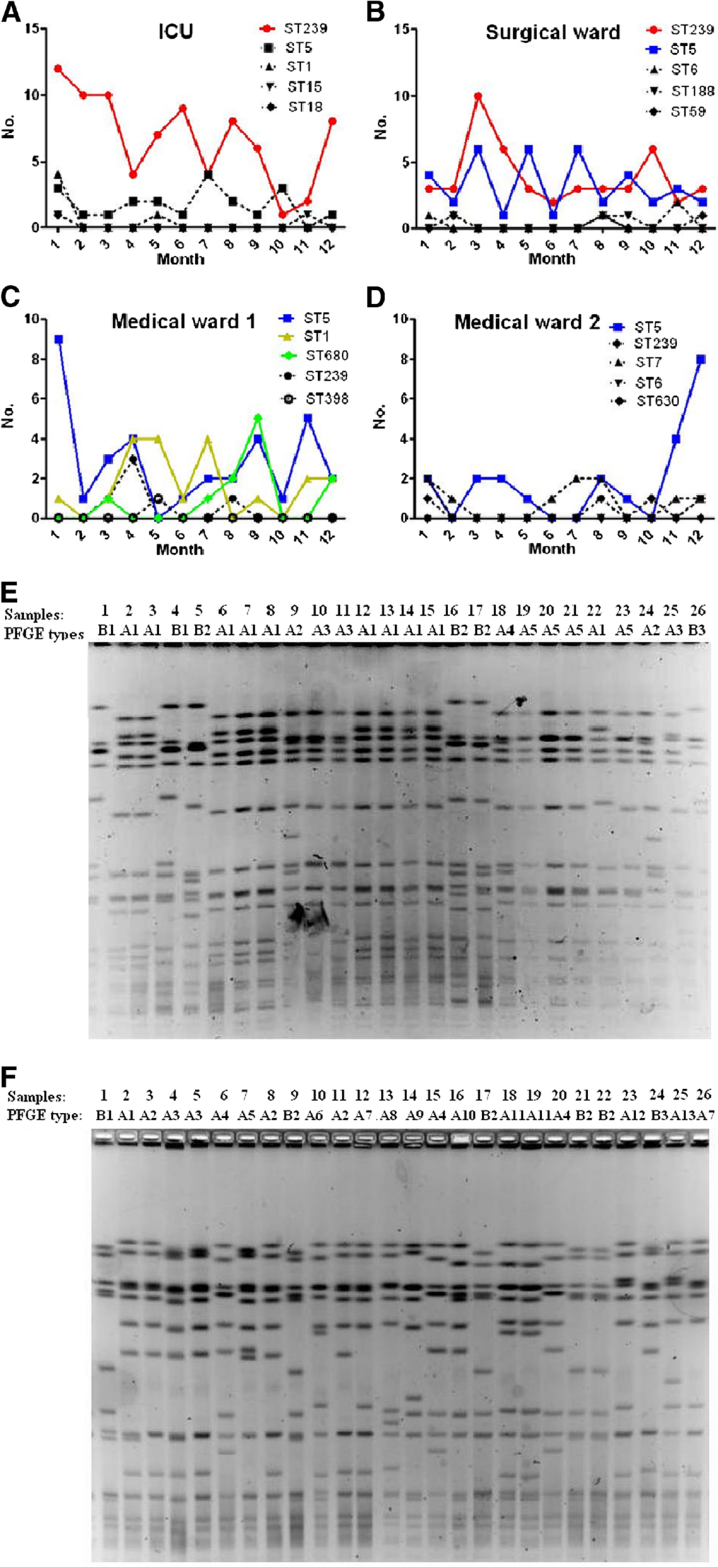
**Dynamic changes of the epidemic *S. aureus *clones in different wards in 2011. A-D**: Dynamic changes of the top five most prevalent *S. aureus* clones in the ICU **(A)**, the largest comprehensive surgical ward **(B)**, and two large medical wards **(C** and **D)**. **E-F**: PFGE profiles of the dominant STs recovered from the same wards. The PFGE profiles of ST239 recovered from the ICU **(E)**. The PFGE profiles of ST5 recovered from medical ward 1 **(F)**. The major DNA restriction pattern was named type A, and isolates with closely (1–3 fragment differences) or possibly related (4–6 fragment differences) restriction patterns were considered subtypes of A, and were designated type A1, type A2, and so on. Those with more than six fragment differences were regarded as type B.

To investigate whether there were epidemic *S. aureus* clones that could survive and spread in different wards, we next analyzed the ICU, one of the largest comprehensive surgical wards, and two of the largest medical wards. As shown in Figure 3, different STs were detected in different wards, and each ward had its own dominant STs. ST239 was a robust sequence type, and was prevalent in the ICU and surgical ward, while ST5 was prevalent in both medical wards and surgical wards. In medical wards, ST5, ST1, and ST680 were the predominant three clones, whilst isolates belonging to other STs were recovered at a rate of three isolates per month. Pulsed-field gel electrophoresis (PFGE) was used to compare the genetic variation of the dominant STs recovered from different wards. Figure 3 (E and F) showed that the restriction profiles of the same epidemic *S. aureus* clones originating from the same wards were not identical. The major DNA restriction pattern was named type A, and isolates with closely (1–3 fragment differences) or possibly related (4–6 fragment differences) restriction patterns were considered subtypes of A, and were designated type A1, type A2, and so on. Those with more than six fragment differences were regarded as type B [13]. PFGE type A1 was the major pattern of the prevalent clone ST239 in the ICU, but the PFGE patterns of prevalent clone ST5 in medical ward 1 were more dispersed.

## Discussion

Surveillance data from China suggested that *S. aureus* infections account for a substantial burden of disease [6]. Most of the individuals infected with hospital-onset *S. aureus* in this study were men (66.0%), which was consistent with findings from a previous study [14]. Unlike the incidence of community-onset *S. aureus*, which is highest in the younger age groups [15,16], hospital-acquired *S. aureus* usually causes infections in immunocompromised populations aged 55 years or above. In our study, more than 60% of *S. aureus* isolates were isolated from this group, suggesting that the biology and pathogenesis of community-acquired *S. aureus* differs from that of hospital-acquired *S. aureus*. Since the 1980s, MRSA has become a serious clinical problem worldwide. In Shanghai, the mean prevalence of MRSA was over 80% in 2005 [4]. Therefore, it is very important to restrict the spread of MRSA in both hospitals and community settings. To control MRSA transmission, measures such as universal hand hygiene practices were introduced into Shanghai teaching hospitals in 2008. This study demonstrated that MRSA healthcare-onset infections were still extremely frequent (68.1%) in the central teaching hospital in Shanghai in 2011, despite the application of infection control measures.

Previous data demonstrated that the epidemic MRSA clones in Asian countries belong to CC8 (ST239) and CC5 (ST5). The ST239 MRSA clone was first discovered in Brazil and has since become widely disseminated in various hospitals [17]. ST239 has been the dominant clone in most of the cities in China since 2005 [18]. In our study, ST239-SCC*mec*III still presented as the most frequent MRSA ST, with ST5-SCC*mec*II identified as the second most common epidemic MRSA clone. This clone was initially described as the main clone in the United States [19] and Japan [20], and was subsequently detected in China [18]. ST239-SCC*mec*I, ST239-SCC*mec*II, ST5-SCC*mec*III, and ST5-SCC*mec*IV were also detected in this study. The occurrence of different SCC*mec* types in the same MRSA clonal lineage led to the hypothesis that these elements were acquired independently at several times through horizontal gene transfer [21]. Multidrug-resistant clones ST239 and ST5 mainly caused respiratory-related infection in our study. This could explain why 78.3% of isolates recovered from patients with respiratory infections were MRSA. ST239 strains were isolated at a frequency of only 8.1 and 3.7% from skin/soft tissue and blood, respectively. ST5 strains were isolated from 16.3% of skin/soft tissue samples in this study, which was lower than in the study of Yu et al. [22], who demonstrated that ST239 strains accounted for only 18.9% of bloodstream infections. We found that ST5 isolates were more susceptible to rifampicin and sulfamethoxazole plus trimethoprim, but more resistant to fosfomycin, than ST239 strains. This implies that appropriate drug selection based on different MRSA types may reduce the reservoir of drug-resistant bacteria. Different STs were associated with different virulence profiles, and the expression of core genome-encoded virulence genes differed between discrete molecular types of *S. aureus*[10,11]. This could explain in part why different clonal types may be associated with specific infection types. Li et al. [23] recently identified a novel surface-anchored protein, SasX, as a crucial factor in promoting nasal colonization, immune evasion, and virulence, and as a probable main driving force of the Asian ST239 MRSA epidemic. Therefore, possible mechanisms for the ST5 MRSA epidemic in this region should be assessed in future studies.

The other two common MRSA STs were ST1-SCC*mec*IV and ST59-SCC*mec*IV, which closely resemble those of the well-known epidemic CA-MRSA clones. ST1 bears the same ST as MW2 (USA400, SCC*mec*IV), which was the first CA-MRSA strain reported in the United States [19]. The major Asian CA-MRSA strain was ST59-SCC*mec*IV [24,25], and was reported to be prevalent in skin and soft tissue infections. The molecular characteristics of the MSSA isolates were genetically diverse in this study, and most MSSA strains caused skin/soft tissue infection and bacteremia. ST7 and ST188 were the two dominant types. ST188 was a double-locus variant of ST1, which was the predicted founder of the community-acquired ST1 type. Sixteen animal-associated clone types, including 15 ST398 and one ST9, were also found in the present study. Human infections caused by ST398 isolates have been reported in many countries [26,27]. All of the ST398 isolates in this study were MSSA, and four carried the gene coding for PVL. PVL is suggested to be an important virulence factor in CA-MRSA isolates, and there is a strong epidemiological association between PVL genes and successful CA-MRSA lineages, especially in skin/soft tissue disease [28,29]. Our data suggest that the external community acted as a significant reservoir of MRSA/MSSA strains related to the skin/soft tissue disease that occurred in hospitals. For this reason, traditional infection control strategies aimed solely at the prevention of MRSA/MSSA transmission in hospitals may be ineffective. New approaches, including public health measures that focus on the community as a source of MRSA/MSSA, are needed to control this epidemic.

In 2008, infection control measures were introduced into Shanghai teaching hospitals to help control the spread of MRSA. Surface-active antiseptics such as chlorhexidine were strongly recommended as decolonization agents in our hospital, especially in the ICU and surgical wards. Emerging resistance to the use of these kinds of antiseptics was a particular concern. The *qacA/B* genes were found in 11.8% of the *S. aureus* clinical isolates in our study. Most of the *qacA/B*-positive clones were MRSA ST239 and ST5, which are very prevalent in the ICU and surgical ward, suggesting that the over-use of antiseptic agents has led to the emergence of MRSA strains with decreased antiseptic susceptibility. Mupirocin treatment was another comprehensive strategy in reducing *S. aureus* colonization and infection in the hospital [30]. In our study, 9.9% of isolates were *muPA*-positive, and the majority of *muPA* -positive isolates were MRSA types ST1 and ST5. Mupirocin resistance in *S. aureus*, especially in MRSA, has been reported in many studies [31,32]. McNeil et al. showed that 11% of *S. aureus* isolates from children with recurrent skin and soft tissue infections carried *muPA*. Thus, detection of mupirocin resistance in *S. aureus*, particularly in MRSA, is necessary to maintain the usefulness of this agent for the treatment of *S. aureus* infections and for infection control.

The rates of hospital-acquired *S. aureus* infection varied between the different departments of Huashan Hospital. During the 12 months of this study, 4198 patients were hospitalized in the ICU for an aggregate of 33,584 days, sustaining 131 hospital-acquired *S. aureus* infections. The rate of hospital-acquired *S. aureus* infection was 3.9 per 1000 ICU-days. The other 31,147 patients were hospitalized in different wards for an aggregate of 386,029 days, sustaining 477 hospital-acquired *S. aureus* infections. The overall rate of hospital-acquired *S. aureus* infection in the other wards was 1.2 per 1000 hospitalized days. Therefore, hospital-acquired *S. aureus* infections in the ICU of the Shanghai teaching hospital pose a greater threat to patient safety than those in the other wards.

Finally, we found each ward had its own dominant STs. This is possibly because different STs exhibit distinct virulence profiles, and each ST is related to specific infection types. In this study, we observed that the strains with the same MLST types did not necessarily have the same PFGE profiles. PFGE can detect genetic variation that accumulates relatively rapidly, and even minor genetic changes (for example, a point mutation resulting in creation or loss of a restriction site) can produce a three-fragment difference in the PFGE gel banding pattern [13,33]. Insertions, deletions, or the presence of plasmids can alter the PFGE pattern without necessarily changing the DNA sequence of the seven housekeeping genes used for MLST, creating diversity in PFGE patterns in the face of homogeneity among MLST patterns obtained for the same isolates. From this point of view, PFGE is more informative than MLST as it involves random screening of the entire genome, whereas MLST analysis is limited to nucleotides within the targeted genes.

## Conclusion

Overall, the present data indicate that there is still a high prevalence of MRSA infections in the teaching hospital in Shanghai, China. The current infection control measures have failed to reduce rates of MRSA infections to acceptable levels for decolonization. The high proportion of multidrug-resistant and chlorhexidine-based antiseptic-resistant clones ST239 and ST5 in the ICU and surgical wards supports the need for more effective infection control measures to curtail the colonization and dissemination of MRSA to hospitalized patients.

## Methods

### Bacterial isolates

From January to December of 2011, 608 sequential *S. aureus* isolates, which represent all the non-duplicate strains isolated during the study period, were collected from inpatients of a comprehensive teaching hospital in Shanghai, China (Huashan Hospital, affiliated with Fudan University). This hospital is located in the center of Shanghai and is one of the largest (1300 beds) teaching hospitals, handling about 8000 admissions per day. All of the inpatients in our study acquired *S. aureus* infection after hospital admission. These isolates were derived from diverse clinical specimens, including the respiratory tract (nasopharyngeal swab and bronchial alveolar lavage fluid), skin and soft tissue (cutaneous abscess and wound secretion), sterile body fluids (pleural cavity fluid, cerebrospinal fluid, and articular cavity fluid), blood, and urine (Table 1). *S. aureus* isolates were confirmed by classic microbiological methods: Gram stain and catalase and coagulase activity on rabbit plasma. *S. aureus* strains were further identified by biochemical characterization using the Api-Staph test (bioMérieux, Lyon, France). All strains were stored at −70°C until use. Research carried out on patients with *S. aureus* infections in accordance with the protocols approved by the ethics committees of Huashan Hospital, Fudan University, Shanghai, People’s Republic of China (Reference number: 2012 M-0072).

### Antimicrobial susceptibility testing

The standard disk diffusion method was used to test the antibiotic susceptibility of all isolates, and results were interpreted in accordance with the Clinical and Laboratory Standards Institute (CLSI) guidelines (CLSI, 2008). Antibiogram classifications were made on the basis of susceptibility to 13 antimicrobials: penicillin(P), levofloxacin (LEV), gentamycin (CN), cefoxitin (FOX), cefazolin (CZ), erythromycin (E), clindamycin (DA), rifampicin (RD), sulfamethoxazole + trimethoprim (SXT), fosfomycin (FOS), teicoplanin (TEC), vancomycin (VA), and linezolid (LZD).

### MLST

Isolates were screened using a previously described method [34] to detect the following seven housekeeping genes: carbamate kinase (*arcC*), shikimate dehydrogenase (*aroE*), glycerol kinase (*glp*), guanylate kinase (*gmk*), phosphate acetyltransferase (*pta*), triosephosphate isomerase (*tpi*), and acetyl coenzyme A acetyltransferase (*yqiL*). The sequences of the PCR products were compared with the existing sequences available from the MLST website (http://www.mlst.net) for *S. aureus*[35], and the allelic number was determined for each sequence.

### PFGE

PFGE was used to compare the genetic diversity of the dominant STs recovered from the same ward. Briefly, *Sma*I-digested DNA embedded in agarose plugs was subjected to PFGE analysis at 14°C in a CHEF-MAPPER system (Bio-Rad) at 6 V/cm, in 0.5 × Tris-borate-EDTA buffer, for two stages: first stage, initial pulse, 5 s; final pulse, 15 s for 10 h; second stage, initial pulse, 15 s, final pulse, 60 s for 10 h; angle 120°.

### SCC*mec* typing

Typing of the SCC*mec* cassette was performed by PCR as described by Kondo et al. [36] and was based on a set of multiplex PCRs (M-PCRs). In this system, M-PCRs 1 and 2 were used for SCC*mec* type assignment; M-PCR 3 or M-PCR 4 was used for J1 region difference-based subtyping, and M-PCRs 5 and 6 were used to identify the integrated copies of transposons (Tn*554* or Tn*554*) and plasmids (pUB110 or pT181).

### *pvl*-, *muPA*-, and *qacA/B*-specific PCRs

Isolates were tested for the presence of the Panton-Valentine leukocidin gene (*pvl*), mupirocin-resistance protein-encoding gene (*muPA*), and chlorhexidine-based antiseptic resistance loci (*qacA/B*) by PCR using the following primers: pvl-F 5′-ATCATTAGGTAAAATGTCTGGACATGATCCA-3′, pvl-R 5′-GCATCAACTGTATTGGATAGCAAAAGC-3′ (PCR product size: 433 bp); *muPA*–F 5′-CATTGGAAGATGAAATGCATACC-3′, *muPA*–R 5′-CGCAGTCATTATCTTCACTGAG-3′ (PCR product size: 443 bp); *qacA/B-*F 5′-CTATGGCAATAGGAGATATGGTGT-3′, *qacA/B-*R 5′-CCACTACAGATTCTTCAGCTACATG-3′ (PCR product size: 416 bp). The amplification was carried out on a GeneAmp 9700 thermal cycler (Applied Biosystems, NY, USA) under the following conditions: an initial 5 min denaturation at 94°C, followed by 35 cycles of 30 s at 94°C, 30 s at 55°C, and 30 s at 72°C, with a final extension at 72°C for 7 min. In each PCR, a positive control and a negative control (distilled water) were included. The PCR fragments were visualized by agarose gel electrophoresis and ethidium bromide staining.

### Statistical analysis

Statistical analyses were performed using Stata software (version 10.1/SE, Stata Corp, College Station, TX, USA). We used the *χ*^2^ and Fisher’s exact tests, as appropriate for analysis of categorical data. Statistical significance was set at *P* ≤0.05.

## Competing interests

The authors declare that they have no competing interests.

## Authors’ contributions

TL performed all experiments. YS assisted in antimicrobial susceptibility testing and YZ in MLST experiment. ML and XD conceived the study and analyzed the results. ML supervised the study and wrote the manuscript. All authors read and approved the final manuscript.
